# Longevity in a Patient With Hypertrophic Cardiomyopathy, Anomalous Coronary Artery, and Gastrointestinal Bleeding

**DOI:** 10.7759/cureus.9677

**Published:** 2020-08-11

**Authors:** Rahul Myadam, Anna Grodzinsky, Randall Thompson

**Affiliations:** 1 Internal Medicine, University of Missouri Kansas City, Kansas City, USA; 2 Cardiovascular Disease, Saint Luke's Mid America Heart Institute, Kansas City, USA

**Keywords:** sudden cardiac death, hypertrophic cardiomyopathy, anomalous coronary artery, acquired von willebrand syndrome

## Abstract

We report an unusual case of an elderly woman who presented to the hospital with melena of five-day duration. She has a past medical history of hypertrophic cardiomyopathy diagnosed three years before presentation. She was found to have arteriovenous malformations in the stomach and the duodenum, causing gastrointestinal bleeding. An association between hypertrophic cardiomyopathy and arteriovenous malformations in the gastrointestinal tract was felt likely. The patient was treated with beta-blocker therapy. Later, she was incidentally found to have an anomalous right coronary artery. We discussed potential medical and surgical options, and the patient chose to be treated medically. She was successfully treated with beta-blocker therapy with no further gastrointestinal bleeding. Her clinical course was uncomplicated without cardiac arrhythmia, heart failure, or sudden cardiac death.

## Introduction

Sudden cardiac death (SCD) accounts for 300,000 to 400,000 deaths annually in the United States [1]. The risk factors for SCD include coronary artery disease, cardiomyopathy, congenital heart disease, and primary electrophysiologic abnormalities. Although knowledge about mechanisms of SCD is increasing, little is known about predicting cardiac events in patients with these underlying these risk factors. This gap in knowledge is higher in the case of elderly patients who are usually asymptomatic. We present an elderly patient with two common risk factors for SCD, which are hypertrophic cardiomyopathy (HCM) and an anomalous coronary artery. This association is unusual and was rarely described previously [2].

The combination of HCM and gastrointestinal bleeding was previously reported in the literature. Blackshear et al. reported 20 patients with HCM and gastrointestinal bleeding, with 11 women having transfusion dependence [3]. Intravascular turbulence resulting in an acquired Von Willebrand disease type IIA was proposed as a possible explanation for this association, similar to patients with aortic valve stenosis and Heyde’s syndrome. Surgical correction of the left ventricular outflow obstruction was found to reduce bleeding in several case reports/series, further strengthening this association. However, in our patient, older age and associated anomalous coronary artery posed unique challenges. We further outline the management of this dual problem in our case report.

## Case presentation

**Figure 1:**
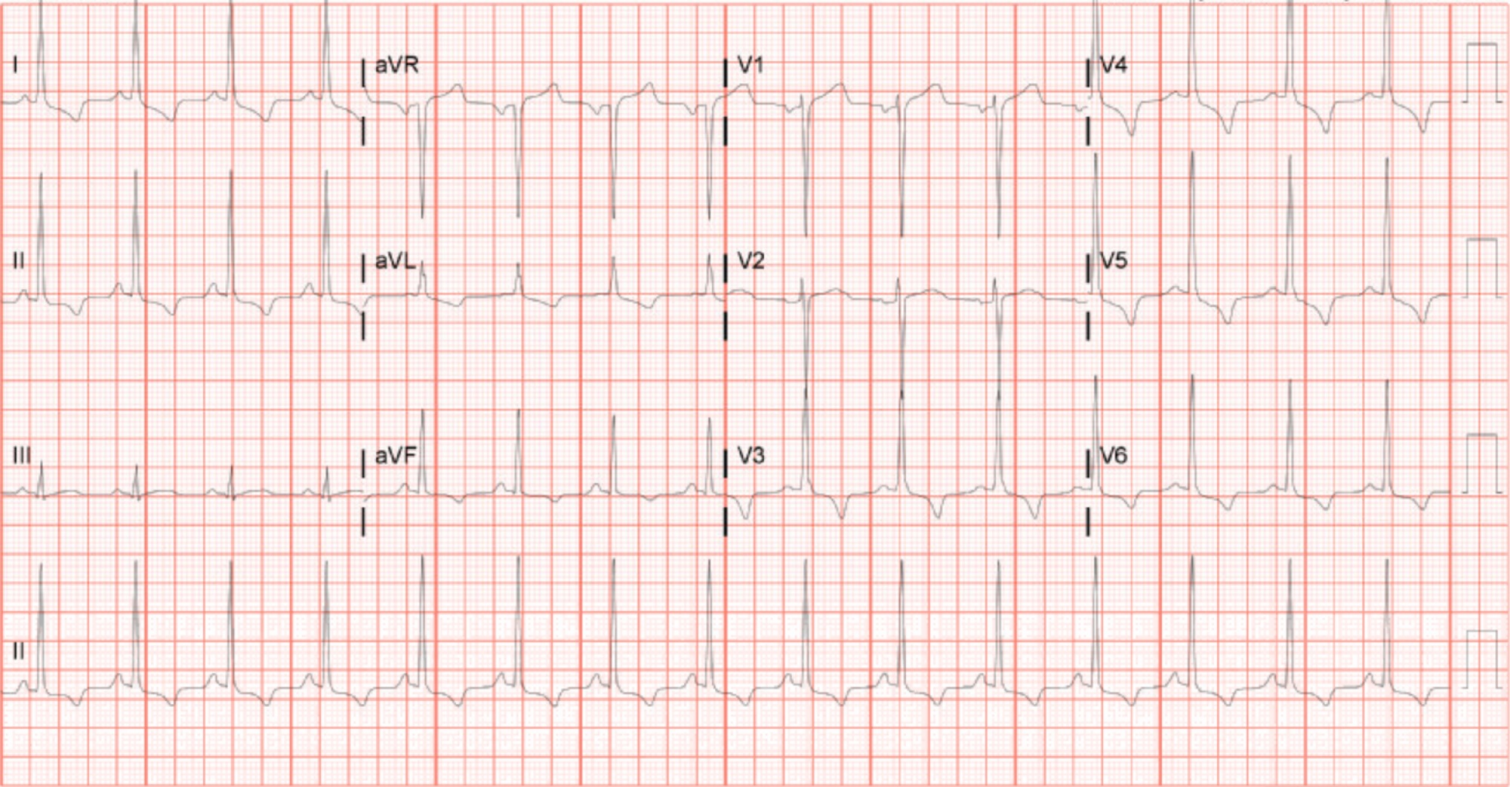
Electrocardiogram showing signs of left ventricular hypertrophy with secondary repolarization abnormalities.

A 73-year-old woman presented to the emergency department with light headedness and black tarry stool for five days. She denied anginal chest pain or syncopal episodes. A 3/6, harsh ejection systolic murmur was best heard in the right upper sternal border. She has a history of HCM diagnosed three years prior. She denied any family history of SCD. EKG (Figure 1) did not show any acute ST-segment changes. Her initial hemoglobin was 5.9 gm/dl (decreased from 10.5 gm/dl two months prior) (normal level 12-15 gm/dl). The first troponin was 1.56 ng/ml and trended up to a peak of 1.95 ng/ml six hours later (normal level <0.01 ng/ml).

**Figure 2:**
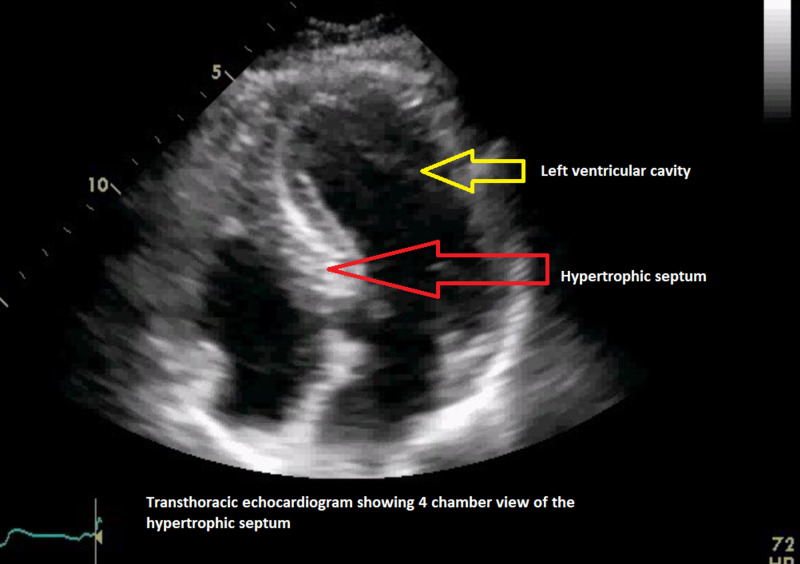
Transthoracic echocardiogram showing hypertrophic septum with a diastolic thickness of 2 cm (normal 0.6-1.1 cm).

Transthoracic echocardiography (Figure 2) showed severe asymmetric left ventricular hypertrophy with systolic anterior motion of the mitral leaflet. There was a dynamic left ventricular outflow obstruction with peak gradient 115 mm Hg (increased from 40 mm Hg two years prior). 

Management

**Figure 3:**
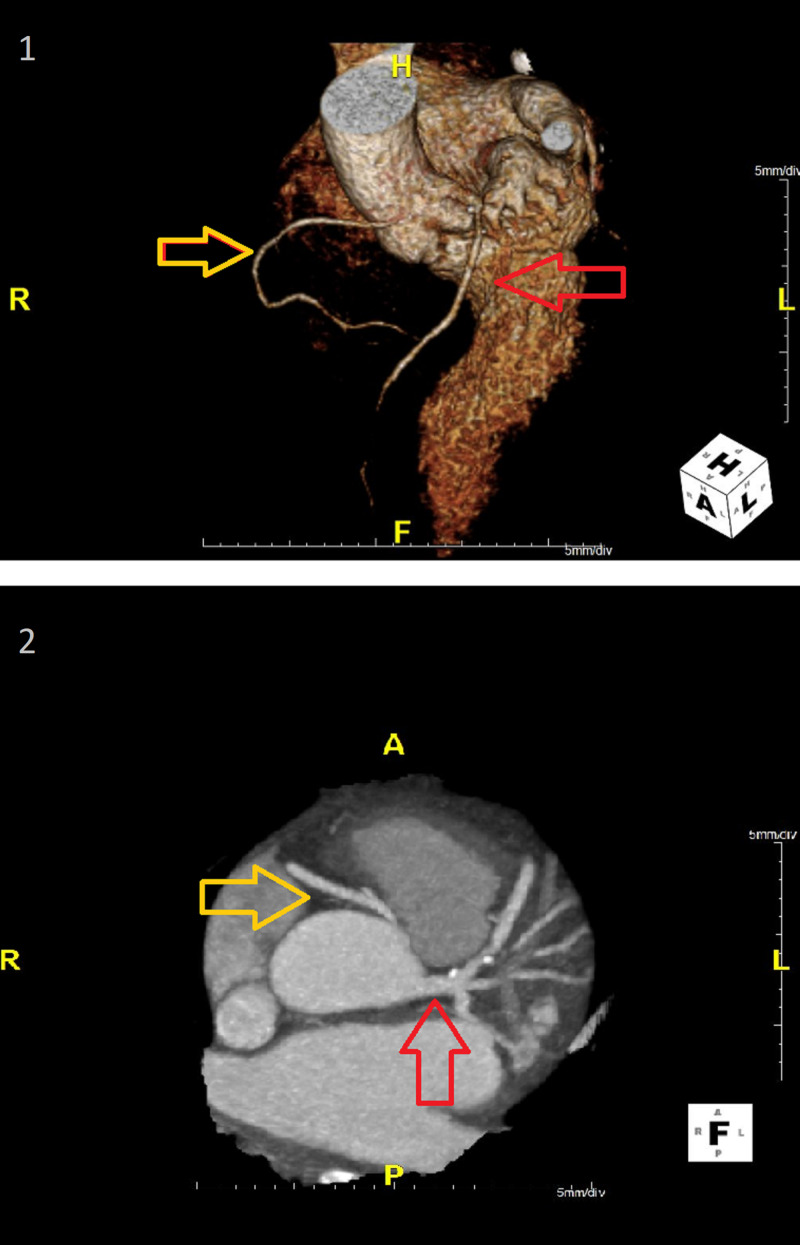
CT scan volume-rendered reconstruction (1) and oblique maximum intensity projection (2) of the coronary arteries. The yellow arrows designate the right coronary artery, and the red arrows the left coronary artery. Both arteries arise from the left coronary sinus.

Upper gastroesophageal endoscopy found angioectasia in the stomach body and duodenal bulb that were treated with thermal therapy. The patient was started on metoprolol 100 mg daily with improvement in hemoglobin. Four months later, she was seen in the cardiology clinic, and a coronary artery x-ray CT scan (Figure 3) was done to evaluate for coronary artery disease. An anomalous origin of the right coronary artery from the left coronary sinus (ARCALS), which coursed interarterially, was diagnosed. There was also a slit-like ostium and severe tubular stenosis in the proximal segment of the right coronary artery. We discussed surgical myomectomy and right coronary artery bypass grafting for management in our patient. However, the patient chose not to undergo an invasive procedure and wanted to be treated medically. An echocardiogram done on medications showed a peak left ventricular outflow gradient improved to 21 mm Hg.

Follow-up

In this context, medical treatment with metoprolol 100 mg daily was continued, and in three years of follow-up, she never experienced any chest pain, syncopal attacks, or heart failure symptoms. We are continuing to follow the patient and adjust further management accordingly closely.

## Discussion

HCM is an autosomal dominant cardiovascular disease, with an estimated prevalence of about 0.2% (1:500) in the United States [4]. The disease has a varied clinical presentation with most affected individuals achieving an average life expectancy. Angelini reported that coronary artery anomalies (CAA) have a global incidence of 5.64% based on a study of coronary angiograms of a continuous series of 1,950 patients [5]. One category of CAA is the anomalous origin of the coronary artery from the opposite sinus (ACAOS), anomalies that are recognized as having severe prognostic implications in young individuals. In a review by Taylor et al. of 242 patients with isolated CAAs, SCD occurred in 13 of 52 patients with ARCALS [6]. However, data in elderly patients are sparse. Rigatelli et al. published that among 5,450 elderly patients (63% above 65 years of age), 66 patients (1.21%) had CAAs [7]. Moreover, most elderly patients with ACAOS are asymptomatic, and these anomalies are usually detected incidentally.

The dual findings of HCM and ARCALS are bothersome because both are independently associated with an increased risk of SCD. Moza et al. described the first case with the two clinical entities in a young man with anginal chest pain and recurrent syncopal attacks [2]. It is not known if the risk of SCD exceeds the combined individual risks from the above conditions. Estimation of risk of sudden death from HCM considers prior personal history of ventricular fibrillation or unexplained syncope, family history of SCD, non-sustained ventricular tachycardia, very thick left ventricular wall (>30 mm maximum thickness), etc. In contrast, risk estimation in patients with ARCALS is unclear. The method for primary prevention of SCD in patients with both lesions is unknown. Placement of an implantable cardioverter-defibrillator was noted by Efthimiadis et al. to be protective. In that case, the patient had a significant risk of SCD based on severe HCM [8]. Our patient had no high-risk features to warrant the insertion of an implantable cardioverter-defibrillator.

The management of ARCALS and HCM should address both problems. The treatment involves either medical (beta-blockers) or surgical therapies. The surgical management of ARCALS involves unroofing, ostial reconstruction, or coronary artery bypass grafting based on the anatomy. Support for medical therapy for ARCALS comes from a large case series published by Kaku et al. that described 56 patients (mean age ~ 56 years) with ACAOS who were treated with beta-blockers and no episodes of SCD reported over five years [9]. In contrast, there is a paucity of long-term follow-up data for surgery to repair ARCALS in elderly patients. Similarly, Heyde’s syndrome from HCM can be treated with beta-blockers or surgical myomectomy. However, a moderately large case series published by Blackshear et al. supports surgical myomectomy or alcohol septal ablation as the preferred treatment [3]. The American Heart Association/American College of Cardiology 2018 guidelines recommend surgery or continued observation for asymptomatic patients with ARCALS if anatomic or physiological evaluation suggests no potential compromise of coronary perfusion [10]. Cheitlin showed that sudden death from ACAOS is seen only in young patients, and the proper management of elderly patients with ARCALS is not precise [11]. In our elderly, sedentary patient, coronary artery surgery seemed challenging to justify, although the calculus would be quite different in a young athlete.

## Conclusions

Our case describes a rare clinical presentation of an elderly patient with HCM and ARCALS who presented with bleeding arteriovenous malformations in the gastrointestinal tract. The optimal management of patients with this dual problem is not well defined and is based on expert advice. In elderly patients, the combined risk of SCD due to HCM and ARCALS is challenging to assess. Our case supports medical therapy with beta-blockers for reducing the risk of SCD in such patients. Our patient's acquired Von Willebrand disease due to HCM was also successfully treated with beta-blockers. 
